# Effects of Shift Work on the Postural and Psychomotor Performance of Night Workers

**DOI:** 10.1371/journal.pone.0151609

**Published:** 2016-04-26

**Authors:** Fernanda Veruska Narciso, José A. Barela, Stefane A. Aguiar, Adriana N. S. Carvalho, Sergio Tufik, Marco Túlio de Mello

**Affiliations:** 1 Universidade Federal de São Paulo, São Paulo, SP, Brazil; 2 Universidade Cruzeiro do Sul, São Paulo, SP, Brazil; 3 Associação Fundo de Incentivo à Pesquisa, São Paulo, SP, Brazil; 4 Universidade Federal de Minas Gerais, Belo Horizonte, MG, Brazil; Oasi Institute for Research and Prevention of Mental Retardation, ITALY

## Abstract

The purpose of the study was to investigate the effects of shift work on the psychomotor and postural performance of night workers. The study included 20 polysomnography technicians working schedule of 12-h night shift by 36-h off. On the first day of protocol, the body mass and height were measured, and an actigraph was placed on the wrist of each participant. On the second day of protocol, sleepiness by Karolinska Sleepiness Scale, postural control by force platform (30 seconds) and psychomotor performance by Psychomotor Vigilance Task (10 minutes) were measured before and after 12-h night work. Results showed that after 12-h night work, sleepiness increased by 59% (p<0.001), postural control variables increased by 9% (p = 0.048), and 14% (p = 0.006). Mean reaction time, and the number of lapses of attention increased by 13% (p = 0.006) and 425% (p = 0.015), respectively, but the mean reciprocal reaction time decreased by 7%. In addition, there were correlations between sleepiness and postural control variables with opened eyes (r = 0.616, 95% confidence interval [CI] = 0.361–0.815; r = 0.538; 95% CI = 0.280–0.748) and closed eyes (r = 0.557; 95% CI = 0.304–0.764, r = 0497; 95% CI = 0.325–0.715) and a pronounced effect of sleepiness on postural sway (R^2^ = 0.393; 95% CI = 0.001–0.03). Therefore, 12-h night work system and sleepiness showed a negative impact in postural and psychomotor vigilance performance of night workers. As unexpected, the force platform was feasibility to detect sleepiness in this population, underscoring the possibility of using this method in the workplace to prevent occupational injuries and accidents.

## Introduction

Night workers are constantly affected by sleepiness and decreased attention and vigilance during and after the night work. Studies have shown that long-term nighttime sleep deprivation and monotonous and prolonged tasks [1, 2] impair the performance tasks that require attention and memory [3, 4], slow the reaction time [5], promote postural instability [4, 6], and increase subjective sleepiness [7]. Sleepiness, sustained wakefulness, total sleep time (TST) of less than 5 to 7 hours, and night work impair overall health, performance during and after labor, and worker safety [3, 4]. Liviya et al. [7] reported a prevalence of 16% of excessive sleepiness among 707 Australian workers. In a study by Scott et al. [8], nurses who worked more than 12 consecutive hours were more susceptible to sleepiness and occupational errors compared with those who worked shorter shifts. In addition, a working time of 40 hours per week had a significant impact on the occurrence of occupational errors. Relevant statistics of occupational and traffic accidents due to sleepiness, night work, and sleep deprivation [9] have concerned researchers and professionals from various sectors. Worldwide, approximately 10 to 30% of fatal traffic deaths are due to sleepiness and fatigue [9]. Fatigue, sleepiness, and risk of occupational injuries and accidents have been associated with long working hours (≥40 hours week), ≥2 consecutive working hours without rest intervals, night work per se, and shift of ≥8 hours [10, 11].

For these reasons, several studies have emphasized the use of specific and sensitive tests for the measurement of sleepiness, hours of wakefulness, and the states of attention and vigilance during and after the shift. The tests commonly applied in the workplace include questionnaires, scales and objective response instruments, such as the psychomotor vigilance task (PVT). The PVT measures the ability to remain alert, aware, and vigilant through responses to stimuli coming from a visual signal [12]. This instrument is considered easy to transport and sensitive for the detection of sleepiness, fatigue, and sleep restriction/deprivation [4, 13]. However, the PVT that can be completed in 10 minutes (PVT-10 min) is considered too long for its application in the workplace because it can primarily affect the logistics of entry and exit hours related to night workers’ shifts. In contrast, the measurements performed using shorter PVT (PVT-90s) can mitigate these logistical difficulties but is considered less sensitive to detect hours of wakefulness and sleep loss [14, 15]. Although the PVT has been previously validated for the measurement and prediction of sleepiness, alertness, and attention of shift workers [12, 13, 16], the importance of diagnosing sleepiness, one of the adverse consequences of shift work, using other methods that are faster and capable of yielding significant results for the promotion of safety in the workplace is underscored. Recently, studies on postural control using posturography reported interesting results on sleepiness, sleep deprivation, and sustained wakefulness. Moreover, sleep deprivation studies have indicated that the measurement of the center of pressure (COP) using posturography over 30 seconds could estimate the hours of wakefulness, sleepiness, and alertness [17–19]. Other studies have demonstrated that sleep deprivation and sleepiness affect postural performance and the integration of systems involved in the postural control, generating increased postural sway [20, 21]. On the basis of this evidence, there is great concern about night workers in relation to the latter part of the shift, particularly between 4 a.m. and 6 a.m. because in this period, humans experience the increased sleepiness, and the worst postural and psychomotor performance [1, 5, 13, 22].

It is important to emphasize that no method is more important than another because the PVT measures variables related to cognition, whereas the analysis of postural control using a force platform measures sensorimotor parameters in behavioral tasks performed daily. Therefore, the possibility of using a fast, practical and sensitive method to measure sleepy or inattentive workers in the workplace further reinforces the intention to prevent accidents and to maintain worker safety during and after the night work. Therefore, the objective of this study was to measure the effects of shift work on the psychomotor and postural performance of night workers.

## Material and Methods

This study was submitted to and approved by the Human Research Ethics Committee of the Universidade Federal de São Paulo under protocol no. 195.746/2013. All of the participants signed an informed consent form after being informed about the study’s objective and procedures.

### Study population

**Fig 1:**
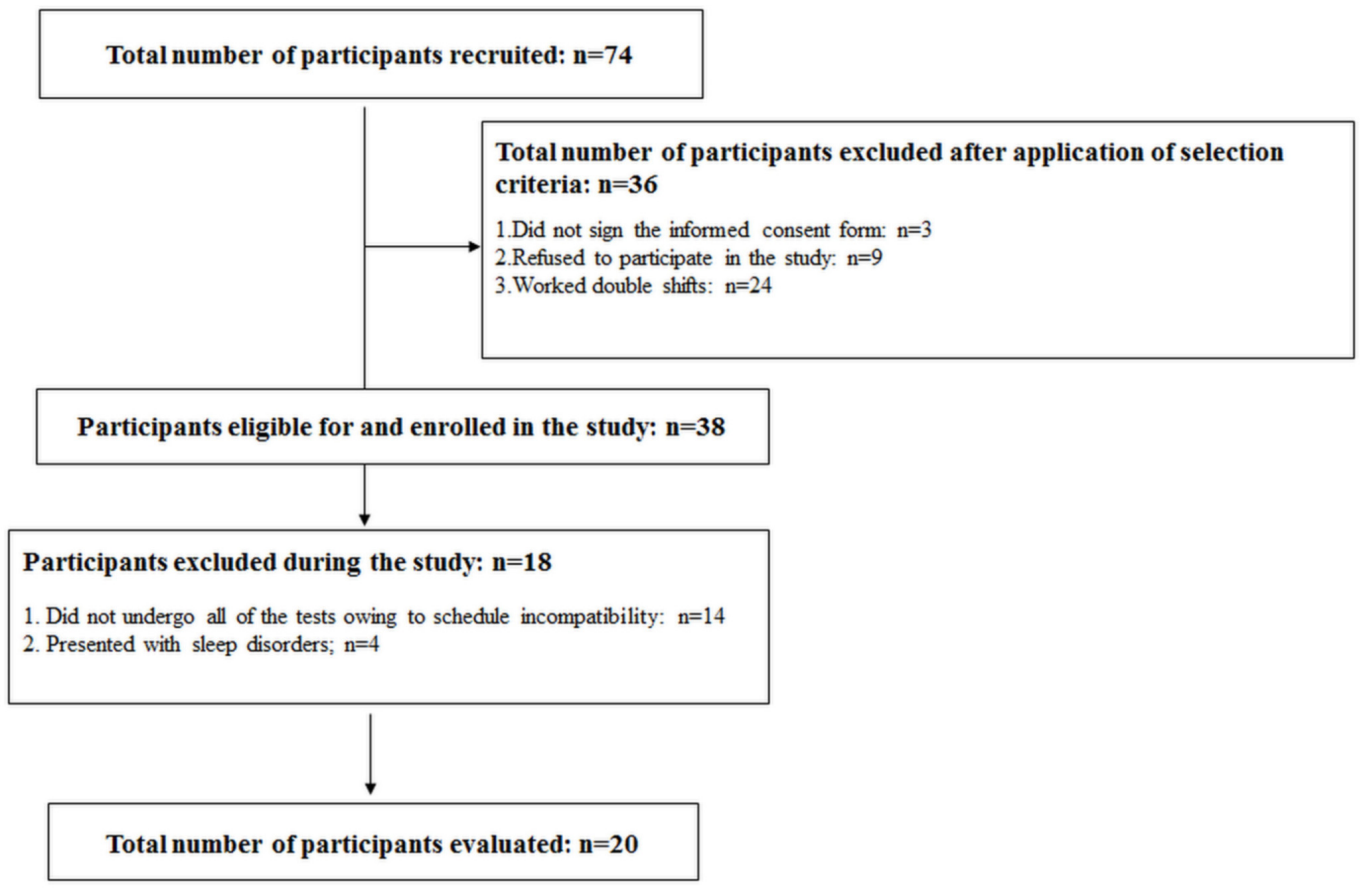
Flow chart of participation in this study.

Of 74 participants, 20 completed the study (Fig 1). The sample consisted of 20 polysomnography (PSG) technicians working night shifts, with a working schedule of 12-h night shift by 36-h off (12 working hours from 8 p.m. to 8 a.m. and 36 working hours off). The participants selected were of both genders, and resided in São Paulo city. All of them were sedentary, were aged between 25 and 55 years, and had worked the night shift for at least one year. The participants were excluded if they worked daytime shifts and double shifts (e.g., morning/afternoon, afternoon/night); napped or slept during the shift; used alcohol 24 hours prior to the measurements; had sleep disorders, hearing and visual impairments, neurological and degenerative brain diseases, or limb amputations; used prostheses; had loss of consciousness; used a wheelchair; had musculoskeletal injuries; had experienced changes in the vestibular system; and did not sign the consent form.

### Procedures

The measurements were performed over two days (days 1 and 2). On day 1, the study participants filled out an individual identification form containing personal and health-related data, received an actigraph, which was placed on their non-dominant wrist, and retained this device until the end of the measurements (after the night work on day 2). The participants were monitored through actigraph and filled out a sleep diary with information on their activity and rest for 5 days. In addition, weight and height were measured, and the body mass index (BMI) was used to assess nutritional status.

The measurements on day 2 began three days after placement of the actigraphs and were conducted in the pre-night shift (after the 36-h working hour off) and post-12-h night shift. The participants were measured approximately 50 minutes before and after the night work (from 7 p.m. to 7:50 p.m. and from 8:10 a.m. to 9 a.m.) in their own work environment. Sleepiness was determined using the Karolinska Sleepiness Scale (KSS), psychomotor vigilance performance was measured using a portable PVT model 192 (Ambulatory Monitoring, Inc., NY), and postural control was measured using an AMTI force platform model OR6 (Advanced Mechanical Technology, Inc., USA).

#### Anthropometric data

Weight and height were measured through a Filizola^®^ anthropometric scale and a stadiometer of the same brand attached to this scale. The participants were asked to remove their personal objects during the measurements (e.g., mobile phones, watches, wallets, etc.) and to remain in a correct standing position on the scale, barefoot, and with arms resting at their sides.

#### Actigraphy

A Sleepwatch actigraph (Ambulatory Monitoring, Inc., NY) was used to calculate the total hours of wakefulness and rest, and the presence or absence of naps during the night shift. The actigraph is a validated and reliable instrument to measure the circadian rhythm, the pattern of rest and mobility, and the sleep-wake cycle in diverse populations [16, 23]. During the measurements, the participants were instructed to press an event marker button on the following occasions: at bedtime, upon awakening, and at the times of removal and replacement of the device.

#### Karolinska Sleepiness Scale (KSS)

The KSS is an instrument used for the subjective measurement of sleepiness, is sensitive enough for the detection of sleep deprivation, and is highly correlated with physiological and behavioral indicators of sleepiness and performance [1, 24, 25]. The KSS originally features nine response scores: 1. “Extremely alert”; 2. “Very alert”; 3. “Alert”; 4. “Fairly alert”; 5. “Neither alert nor sleepy”; 6. “Some signs of sleepiness”; 7. “Sleepy, but no effort to keep alert”; 8. “Sleepy, some effort to keep alert”; 9. “Very sleepy, great effort to keep alert, fighting sleep” [24]. KSS scores of ≥7 were correlated with electroencephalographic signals of objective sleepiness [25].

#### Psychomotor Vigilance Task (PVT)

The PVT-10 min was used to measure sustained attention and psychomotor vigilance of participants [12, 26]. The protocol adopted used only visual response tests [4] in which bright red visual stimuli (from a light-emitting diode [LED] digital counter) were flashed at intervals of 2 to 10 seconds on the screen of the device [12]. The participants were asked to press a response button, located on the right side of the device, as soon as the visual stimuli appeared. In this study, the variables analyzed included mean reaction time (mean RT), mean reciprocal reaction time (mean RRT), and number of lapses of attention. The values obtained were analyzed using the software React (Ambulatory Monitoring, Inc., NY).

#### Postural control

The postural control consisted of measurements of postural sway by means of the calculation of the COP in the standing position on a force platform. To conduct these tests, the participants were instructed to remain in an upright position as stable as possible, barefoot, with arms resting at their sides, on a bipedal support base, with eyes opened or closed. A total of two tests were conducted, with two replicates and a 1-min interval between each replicate [27]. Each measurement of postural control on the force platform lasted 30 seconds [18, 27, 28], the participants were instructed to stare at a black target approximately 5.0 cm in diameter, positioned on a white wall at eye level at a distance of 2.0 meters. The reaction force and moments of force on the force platform were acquired using an acquisition card (NI BNC-2090, National Instruments, Inc.) at a frequency of 100 Hz [27, 28]. To measure the COP in the anterior-posterior and medial-lateral directions, the software LabView (National Instruments, Inc.) was used. Subsequently, COP values were filtered (Butterworth digital low-pass filter, second order, cutoff frequency of 5 Hz), and mean sway amplitude in the anterior-posterior (MSA_(a-p)_) and medial-lateral directions (MSA_(m-l)_) and total sway displacement (TSD) were calculated using specific routines written with MATrix LABoratory (MATLAB) software (The Mathworks, Natick, MA) as previously described.

#### Statistical analysis

Repeated measures Student’s t-test was used to compare the variables sleepiness, mean RT, number of lapses of attention, MSA_(a-p)_, MSA_(m-l)_, and TSD of the COP before and after the night work. Pearson’s correlation coefficient was used to calculate the correlation between the following variables: sleepiness x postural control, sleepiness x psychomotor performance, hours of wakefulness x postural control, and hours of wakefulness x psychomotor performance. Linear regression analysis was conducted to assess the effect of sleepiness on the postural performance of the night workers. Sleepiness was considered the independent variable, and MSA_(a-p)_ was considered the dependent variable. The α-level for all analysis was set at 0.05.

## Results

The study sample consisted of 15 women (75%) and 5 men (25%), all of whom were sedentary, with a mean age of 35.1 ± 7.0 years, and a mean BMI of 26.2 ± 6.3 kg/m^2^. The total hour of wakefulness after the night work was 1242.0 ± 213.73 minutes or 21 hours (Table 1).

**Table 1:** Comparison of the variables related to postural control and psychomotor performance of night workers before and after the night work (n = 20).

Variables	Pre-night shift Mean±SD (95% CI)	Post-night shift Mean±SD (95% CI)	*t*	Effect size	Delta (%)	p-value
Sleepiness -KSS	3.45±1.64 (2.69, 4.17)	5.50±1.91(4.61, 6.32)	5.037	1.2	59%	<0.001*
Hours of wakefulness (min)	485.10±209.80 (395.25, 570.80)	1242.0±213.73 (1150.82, 1330.91)	57.156	3.6	156%	<0.001*
**Postural control (eyes opened)**						
MSA_(a-p)_ (cm)	0.18±0.06 (0.16, 0.21)	0.20±0.06 (0.17, 0.23)	2.110	0.3	9%	0.048*
MSA_(m-l)_ (cm)	0.11±0.05 (0.09, 0.13)	0.12±0.05 (0.10, 0.14)	1.056	0.2	8%	0.304
TSD (cm)	929.98±268.08 (810.20, 1044.29)	1055.93±296.75 (933.73, 1190.46)	3.117	0.4	14%	0.006*
**Postural control (eyes closed)**						
MSA_(a-p)_ (cm)	0.21±0,06 (0.18, 0.24)	0.22±0.06 (0.19, 0.25)	0.951	0.1	4%	0.353
MSA_(m-l)_ (cm)	0.12±0.05 (0.10, 0.14)	0.14±0.05 (0.12, 0.16)	1.535	0.3	12%	0.141
TSD (cm)	965.05±268.31 (857.88, 1088.53)	1113.88±398.57 (960.00, 1315.64)	2.115	0.4	15%	0.048*
**Psychomotor performance—PVT**						
Mean RT (ms)	250.02±31.86 (236.79, 265.09)	281.45±60.93 (257.46, 312.61)	3.077	0.7	13%	0.006*
Mean RRT (ms)	4.23±0.42 (4.05, 4.40)	3.93±0.59 (3.67, 4.19)	3.937	0.6	7%	0.001*
Number of lapses of attention	0.60±0.75 (0.28, 0.95)	3.15±4.61 (1.33, 5.55)	2.567	1.0	425%	0.019*

* Significant difference when comparing the variables before and after the night work (p<0.05). Repeated measures Student’s t-test was performed.

After the night work, the mean of sleepiness and hours of wakefulness increased by 59% and 156%, respectively, indicating an increase in sleepiness during and after the 12-h night work. A decreased by 7% in mean RRT was observed, but the mean RT, and the mean number of lapses of attention increased by 13% and 425%, respectively after 12-h night work. The mean postural sway increased with eyes opened (MSA_(a-p)_ = 9%; TSD = 14%) and eyes closed (TSD = 15%) after 12-h night work (Table 1). However, MSA_(m-l)_ with eyes opened and MSA_(a-p)_ and MSA_(m-l)_ with eyes closed did not increase significantly after the 12-h night work (Table 1). Therefore, after the night work, there were significant increases in sleepiness, hours of wakefulness, postural sway, and a decrease in psychomotor performance.

Our results indicated a moderate positive correlation between sleepiness and the postural control variables with eyes opened: MSA_(a-p)_ (r = 0.616; CI_95%_ = 0.361–0.815) and TSD of the COP (r = 0.538; CI_95%_ = 0.280–0.748); and with eyes closed: MSA_(a-p)_ (r = 0.557; CI_95%_ = 0.304–0.764) and TSD of the COP (r = 0.497; CI_95%_ = 0.325–0.715). Therefore, the higher the level of sleepiness, the higher the postural sway. However, no significant correlations were observed between sleepiness and MSA_(m-l)_, mean RT, mean RRT, and number of lapses of attention; similarly, no significant correlations were observed between the hours of wakefulness and psychomotor performance variables (mean RT, mean RRT, and number of lapses of attention), and postural sway with eyes opened and eyes closed (MSA_(a-p)_, MSA_(m-l)_, and TSD) (Table 2).

**Table 2:** Correlation coefficients (*r*) of sleepiness and hours of wakefulness for the variables related to psychomotor performance and postural control (n = 20).

			Psychomotor performance			Postural control	
		Mean RT	Mean RRT	Lapses of attention	MSA_(a-p)_	MSA_(m-l)_	TSD
						**Eyes opened**	
Sleepiness	*r* (p-value)	0.388 (0.091)	-0.429 (0.059)	0.302 (0.195)	0.616 (0.004)*	0.389 (0.090)	0.538 (0.014)*
Hours of wakefulness	*r* (p-value)	0.367 (0.111)	-0.412 (0.071)	0.326 (0.160)	-0.237 (0.314)	-0.324 (0.058)	-0.287 (0.220)
						**Eyes closed**	
Sleepiness	*r* (p-value)				0.557 (0.011)*	0.407 (0.075)	0.497 (0.026)*
Hours of wakefulness	*r* (p-value)				-0.178 (0.453)	-0.288 (0.218)	-0.096 (0.687)

* Significant difference after correlation of the variables (p<0.05) using Pearson’s correlation coefficient.

**Fig 2:**
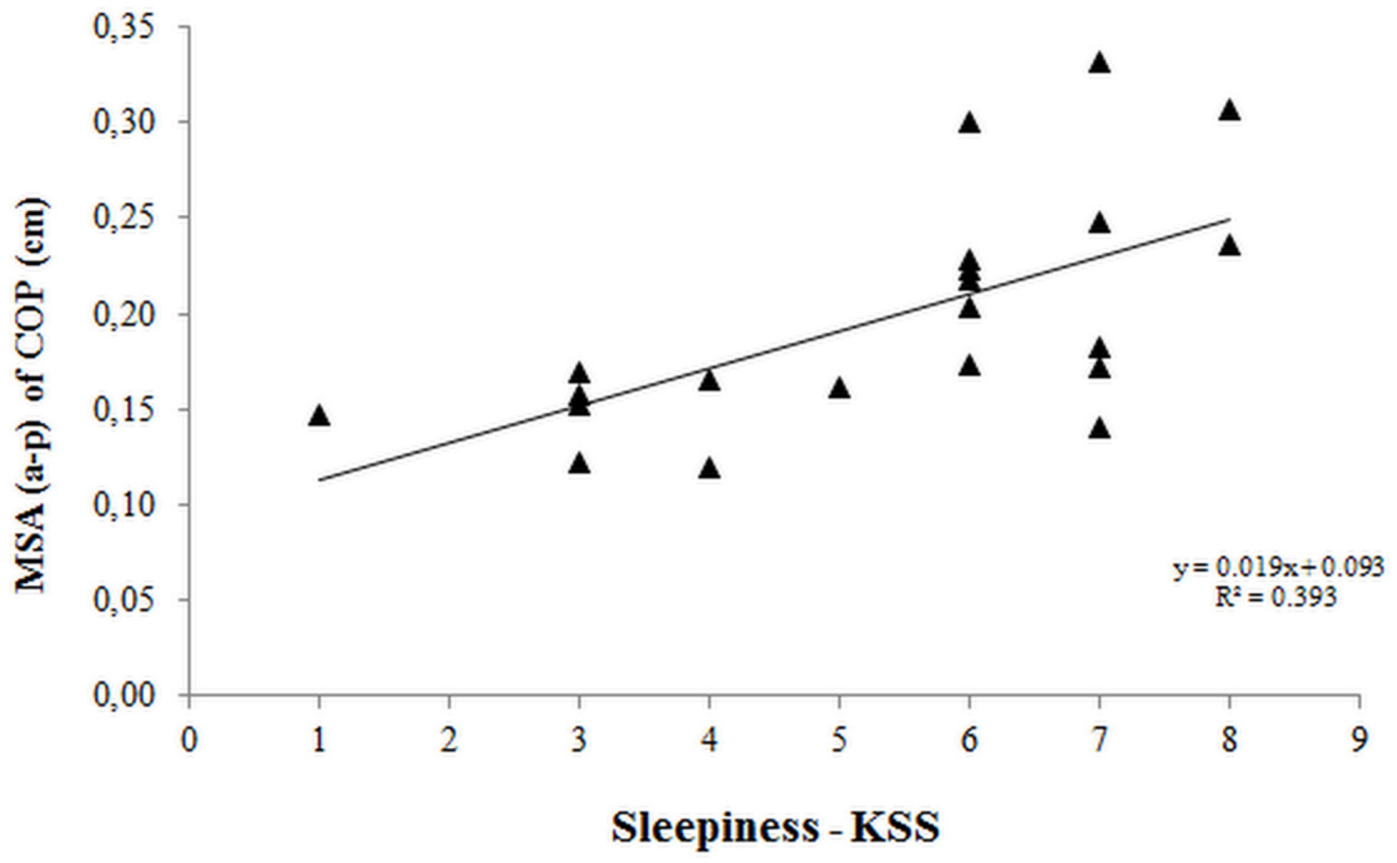
Effect of sleepiness on the mean sway amplitude of the center of pressure in the anterior-posterior direction of 20 night workers assessed by linear regression analysis.

Linear regression analysis indicated that for each increase in the score for sleepiness, postural sway (MSA_(a-p)_) with eyes opened increased 0.02 cm (R^2^ = 0.393). The confidence limits (0.01, 0.03) indicated a 95% confidence that the slope for the population was between these limits, and the F_(1,20)_ test = 10,996 presented an associated probability level of p = 0.004 (Fig 2). Therefore, the increased level of sleepiness significantly affected the postural sway of the night workers, corroborating the worsening of the postural control in this population due to sleepiness, which is one of the adverse effects of night work.

## Discussion

The present study investigated the effects of the shift work on the psychomotor and postural performance of night workers. The results indicated increased sleepiness and decreased postural and psychomotor performance after a 12-hour night shift in agreement with previous studies [18, 29, 30]. When the measurements were analyzed after the night shift, the KSS, number of lapses, mean RT, mean RRT, and postural sway demonstrated that night workers felt less alert, sleepier, and were less vigilant. Similar results were obtained by Geiger-Brown et al. [13], who observed that nurses working 12-h night shifts displayed higher levels of sleepiness at the end of the shift compared with the beginning of the shift and an increase in the number of lapses of attention for every consecutive day of work. Despite the use of different approaches, the increased number of lapses of attention, excessive sleepiness, and the consequent decreases in psychomotor and postural performance associated with the duration of the shift work have been observed in health and transportation professionals [13, 29, 31]. Vetter et al. [29] reported that the median reaction time increased among night shift workers throughout the shift work, and the decreased psychomotor performance was correlated with more than 9 hours of continuous wakefulness. In addition, Harma et al. [30] found that the duration of the shift work increased the risk of severe sleepiness (KSS≥7) by 15% for each hour worked. Therefore, the increase in the duration of the shift from 6 to 9 hours increased the risk of sleepiness in 50% of the workers, demonstrating the effect of shift duration on sleepiness. During the 12-h night shift, a sharp decline in the psychomotor performance of pilots and co-pilots of a Brazilian airline was also reported by de Mello et al. [31]. In this context, it appears that the 12-h night shift combined with subjective sleepiness was an important finding of the present study, and it was possible to observe that psychomotor vigilance and attention were impaired among night workers, with important implications for the risk of occupational errors and an increase in the number of accidents.

Our results indicated that the magnitude and direction of the postural sway (MSA_(a-p),_ and TSD of the COP) of the workers with eyes opened or closed measured for 30 seconds increased after a 12-h night shift. Studies demonstrated that sustained wakefulness and sleep deprivation can decrease the overall metabolism and blood flow in certain brain areas that are important for cognition and performance of high-demand sensorimotor tasks (thalamus, basal forebrain, basal ganglia, and parietal and prefrontal cortices), thereby affecting sensory integration of posture control [32, 33]. Impairment of sensory integration or sensorimotor coupling due to nighttime sleep deprivation produces deleterious effects on postural control, affecting the fine balance to obtain the most relevant sensory stimuli for the maintenance of the desired postural orientation [19]. Another factor is that although visual information is important for stability [2, 20, 32], it was not enough to compensate for the impairment of the functioning of the postural control system of the workers measured caused by night shift. Karita et al. [34] reported that the measurement of COP variables of workers on a force platform could diagnose fatigue due to insufficient sleep and excessive working hours. Other researchers observed increased COP area in adults deprived of sleep for 12 hours (186.50 ± 110.03 mm^2^) compared with those with normal sleep (137.93 ± 66.31 mm^2^) and concluded that a single night of sleep deprivation can impair the integration of sensory systems and cause inefficiency in the vestibular system, regardless of the presence or absence of vision, consequently compromising postural control [22]. The current study also observed an increased frequency of postural sway with eyes closed after 26 hours of sleep deprivation. The authors suggested that the suppression of vision enhances the effect of fatigue and affects postural performance [20]. In agreement with recent laboratory studies, a point to note is that the present study conducted in the workplace confirmed the increases in postural sway with eyes opened and closed as a result of sleep deprivation, and sleepiness, one of the consequences of the shift work [34, 35].

Nevertheless, another result showed that there was no correlation between sleepiness and the variables related to psychomotor performance (mean RT, mean RRT, and lapses of attention). This finding suggests that a possible reason could be that different brain areas or different stimuli pathways are involved in the response of each of these behaviors during the sleep deprivation. Another reason is that there is a main difference between these two types of tasks (subjective alertness and objective vigilance) whom demands different attentional processes [36, 37], therefore, we assume that these physiological factors may have affected the association between the variables, even though the PSG technicians experienced sleepiness and worsening of psychomotor performance at the end of the night shift, as the results indicated. In disagreement with previous findings [20, 38, 39], but not with Sargent et al. [40], we found no correlation between the hours of wakefulness (21 hours) and the postural control parameters in this study, most likely because the previous studies have employed different protocols and measurement parameters (time of the biological day, duration of wakefulness, and total of sleep deprivation), and have reported increased postural sway only after 24 hours of sleep deprivation [6, 21], moreover, few studies have measured these variables at the end of the night shift [18]. Similar to this study, Sargent et al. [40] reported that the postural control is not influenced by hours of wakefulness, indicating that fatigue associated with sustained wakefulness may not be assessing for postural sway parameters.

In contrast, we found an unexpected finding in the present study. There was a moderate correlation between sleepiness and postural control (mean sway amplitude and total sway displacement). Thus, the increased levels of sleepiness resulted in increased postural sway affecting the night workers’ ability to remain stable in the standing position and impairing their motor activities, potentially leading to the increased risk of falls, accidents, and injuries during and after the night work. A similar study, although using other analyses of the COP, reported postural changes in 5 out of the 71 drivers measured after the shift and a strong correlation between sleepiness and worsening of postural control [18]. Therefore, we emphasize the extent to which sleepiness impairs postural stability and possibly the integration of systems involved in postural control. As corroborated in previous studies [18, 21, 35], we emphasize that nighttime sleep deprivation, night work, and sleepiness decrease the ability of individuals to remain vigilant, alert, and stable in standing positions. Therefore, the confirmation that the night work combined with nighttime sleep deprivation increases the magnitude of postural sway corroborates the hypothesis that short-term tests (30s) on a force platform can detect changes in postural sway and adequately measure the levels of alertness and sleepiness in these workers, considering the impairments of psychomotor and postural performance and increased sleepiness during and after the night shift.

Considering the results of this and previous studies, it was possible to demonstrate that the COP variables measured on the force platform and the psychomotor vigilance variables measured using the PVT are sensitive and feasibility of predicting the effects of night work, particularly sleepiness. According to some researchers, the tests performed on a force platform can be easily and quickly applied, have already been validated, and are sensitive enough to measure fatigue and sleepiness as a result of the hours of wakefulness, sleep deprivation, and work conditions [20, 39]. In addition, their application in the work environment aims at the prevention of occupational and traffic accidents due to sleepiness and long shifts (12-h). Therefore, reliable and practical measurement tools and concrete strategies to avoid or mitigate sleepiness should be adopted during and after the shift.

This study had some limitations. The generalization of the results may be limited because of the prevalence of women in the study sample (75%). However, similar results were obtained in studies with a prevalence of men [18, 28], which likely minimizes this limitation. Another limitation was that controls for the circadian period were not used in the measurements because the work schedule was not flexible. Therefore, more studies using different work conditions conducted in the work environment are necessary to confirm the effects of shifts on sensory-motor integration.

In short, our results indicate a worsening of postural control due to the sleepiness experienced by night workers after 12 consecutive working hours, corroborating another of the many deleterious effects of night work reported in previous studies [3, 13, 16].

## Conclusion

The night workers of this study experienced increased subjective sleepiness and consequent decreases in psychomotor and postural performance after consecutive 12-h night shift. The sleepiness, one of the harmful effects of night work as well as sleep deprivation, influenced the postural performance of these workers; accordingly, the force platform was feasibility to detect one of the effects of night work in this worker population, underscoring the possibility of using this method in the work environment to prevent occupational injuries and accidents associated to subjective sleepiness.
